# Associated factors of headache in an unstudied cohort of elderly subjects

**Published:** 2016

**Authors:** Alijan Ahmadi Ahangar, Seyed-Reza Hossini, Farzan Kheirkhah, Ali Bijani, Zahra Moghaddas

**Affiliations:** 1 Mobility Impairment Research Center, , Babol University of Medical Sciences, Babol, Iran.; 2 Social Departments of Health Research Center, Health Research Institute, Babol University of Medical Sciences, Babol, Iran.; 3 Department of Psychiatry, Babol University of Medical Sciences, Babol, Iran.; 4 Babol University of Medical Sciences, Babol, Iran.

**Keywords:** Depression, Elderly subjects, Headache, Pain.

## Abstract

**Background:**

Headache and depression are prevalent among general population. The aim of this study was to determine the associated factors of headache in elderly subjects with emphasis to depression.

**Methods:**

All cohort of elderly individuals of the Amirkola Health Study Project were included. Data regarding several clinical and demographic characteristics were provided via fill in quesstionnaire, interview and clinical examination. Presence and duration as well as severity of headache were collected through an interview based on self-reported data. Diagnosis of depression was confirmed according to standard Geriatric Depression Scale (GDS) criteria. In statistical analyses chi-square test with logistic regression analysis was used for association.

**Results:**

A total of 832 men and 667 women aged >/= 60 years old were studied. Headache and depression were diagnosed in 42% and 42.4% respectively. In depressed subjects, headache was significantly higher by OR=3.1(95% CI, 2.5-3.83, P=0.001). Proportions of headache increased by severity of depression with a dose-response pattern of relationship from 53.3% in mild depression to 72.6% in severe depression. The magnitude of OR for headache increased from 2.59 (95% CI, 2.03-3.31) in patients with mild depression to 6.04 (95% CI, 3.54-10.3) in patients with severe depression. After adjustment for all covariates, headache was significantly associated with female gender and back pain as well as with depression with a significant dose-response relationship.

**Conclusion:**

The findings of this study indicated an independent association between headache and psychological factors in elderly subjects, particularly in women.

**H**eadache and depression are prevalent conditions involving a significant proportion of general population. Depression is particularly a chronic condition with frequent relapsed which often lead to work discontinuation, disability and financial burden (1, 2). However, early diagnosis and treatment may control depression symptoms and prevent future recurrences (3). Clinical manifestations of depression in the elderly population are somewhat different and in spite of the presence of several medical problems, elderly subjects feel dissatisfied (4). Several clinical, biological, and psychological factors as well as chronic pain are associated with depression (3, 5, 6). Patients with persistent chronic pain such as chronic headache in particular older subjects are vulnerable to both depression and high level of stress (7, 8). Several studies have shown a relation between depression symptoms and chronic neck pain back pain, and headache (5-7). The prevalence of chronic pain is two times greater in subjects with depressive symptoms as compared with healthy subjects (5). The prevalence is higher in elderly depressed subjects (9) and treatment of both headache and depression exerted greater benefits (10).

Nonetheless data regarding headache and depression in elderly subjects are scarce (5). This issue is important because many chronic medical conditions like osteoarthritis, diabetes, coronary heart disease are prevalent in elderly subjects. These conditions are usually associated with chronic pain and require persistent conservative medical treatment. This may lead to depression or exacerbation of the underlying depressive syndrome. For these reasons, the present study was designed to determine the associated factors of headache in a cohort of elderly subjects aged 60 years and older.

## Methods

This descriptive analytical cross-sectional study is a part of a comprehensive cohort study of the health status of the elderly subjects of Amirkola. The Amirkola Health and Aging Project. (AHAP no. 892917). Done as a cohort study, started on 2012 on all people of 60 years of age and older and is still currently under process (11).

All inhabitants of Amirkola town aged 60 years and over were invited to participate in this cohort study. Demographic information was collected by questionnaires and in-person interviews. The information on headaches was self-reported, collected from all elderly people individually. Self-reported data were provided in regard to duration of headache, quality and intensity of headache and its influence on daily activities and quality of life (12, 13). The data on depression were provided by using the standard Geriatric Depression Scale (GDS) consisted of 15 questions. Details of data collection have been described elsewhere (11). Patients were classified in 4 groups according to GDS score as normal (0-4), mild (5-8), moderate (9-11) and severe (12-15) (3). Chronic pain was defined as persistent of pain for at least 90 days over six months prior to interview. In statistical analysis chi-square test was used for comparison of proportion as well as association. Logistic regression analysis with calculation of odds ratio (OR) and 95% confidence interval (95% CI) was applied to determine independent association. SPSS software was used for analysis.

## Results

A total of 1499 out of total 1616 elderly persons who nominated for this study were deemed suitable for investigation (832 men and 667 women) the remainders were excluded due to medical conditions. Overall 630 (42%) patients had headache (female 63%) and 637 (42.4%) had depression (female 62.6%) table 1.

As shown in table 1, headache in patients with depression was significantly higher than normal patients (57.6% vs 30.5%, OR=3.1 (95% CI, 2.5-3.83, P=0.001) indicating a significant association between depression and headache.

**Table 1:** Association between headache and depression in in elderly subjects of the Amirkola Health Study Project by calculation of odds ratio (OR) and 95% confidence interval (95%CI

**Study groups**	**No (%)**	**With headache** **No (%)**	**Without headache** **No (%)**	**OR(95%CI)**
Depression	637 (42.5)	367 (57.6)	270 (42.4)	3.1(2.5-3.83)
Healthy	862 (57.5)	263 (30.5)	599 (69.5)	
Total	1499 (100)	630 (42)	869 (58)	

# Compared with chi-square test

As shown in table 2, proportions of headache increased by severity of depression from 53.3% in mild depression to 72.6% in severe depression. The magnitude of OR increased from 2.59 (95% CI, 2.03-3.31) in patients with mild depression to 6.04 (95% CI, 3.54-10.3) in patients with severe depression suggesting a dose-response pattern of relationship between depression and headache. The association between headache and several demographic factors and clinical conditions are presented in table 3. Headache in females was significantly higher than males (OR=3.83 (95% CI, 3.08-4.75, P=0.001) indicating an association of headache with female sex. There were also significant associations between headache and chronic pain (OR=1.83, 95% CI 1.36-2.46), back pain (OR= 2.72 (95% CI, 2.17-3.4). In addition prevalence of headache was significantly higher in single subjects and those who live alone, vitamin D sufficient subjects and patients lower than normal MMSE score (table 3). The results of logistic regression analysis after adjustment for all covariates, demonstrated a significant independent association between headache female sex by OR=2.48 (95% CI 1.94-3.17), back pain by OR= 1.95 (95% CI,1.49-2.53), and depression with a dose – respone relationship pattern by OR=1.94 (95% CI,1.49-2.52) and OR= 2.39 (95% CI,.1.65-3.49) and OR= 3.29 (95% CI, 1.86-5.81) for mild, moderate and severe depression respectively. The association with other variables (table 3) diminished to non-significant levels.

**Table 2:** Association between headache with severity of depression in in elderly subjects of the Amirkola Health Study Project by calculation of odds ratio (OR) and 95% confidence interval (95% CI

**Study groups**	**No (%)**	**With headache** **No (%)**	**Without headache** **No (%)**	**OR(95%CI) ** #
Healthy	862 (57.5)	263 (30.5)	599 (69.5)	1
Mild depression	400 (26.7)	213 (53.3)	187 (46.8)	2.59 (2.03-3.31)
Moderate depression	164 (10.9)	101 (61.6)	63 (38.4)	3.65(2.58-5.1)
Severe depression	73 (4.9)	53 (72.6)	20 (4.27)	6.04(3.54-10.3)
Total	1499 (100)	630 (42)	869(58)	

# Compared with chi-square test

**Table 3.** Association between headache with demographic characteristics and some common clinical conditions in elderly subjects of the Amirkola Health Study Project by calculation of odds ratio (OR) and 95% confidence interval (95% CI

**variable**		**Frequency ** **(%)**	**With headache** **Frequency ** **(%)**	**Without headache** **frequency ** **(%)**	**OR(95%CI) ** @
Age	60-64	533 (36.9)	253 (45.8)	300 (54.2)	1
	65-69	314 (29.9)	118 (36.7)	196 (62.4)	0.69 (.52-.92)
	70-74	264 (17.6)	110 (41.7)	154 (58.3)	0.85 (.63-1.14)
	75-79	229 (15.3)	103 (45)	126 (55)	0.97 (.71-1.3)
	80-84	93 (6.2)	34 (36.6)	59 (63.4)	0.68 (.43-1.08)
	85-99	46 (3.1)	12 (26.1)	34 (73.9)	0.42 (.21-.93)
Sex	female	667 (44.5)	398 (5.7)	269 (40.3)	3.83 (3.08-4.75)
	male	832 (55.5)	232 (27.9)	600 (72.1)	1
Level of education	Illiterate	953 (63.3)	412 (43.2)	541 (56.8)	1
	Elementary	413 ( 27.6)	182 (44.1)	231 (55.9)	1.03 (.82-1.31)
	primary	28 (1.9)	11 (39.3)	17 (60.7)	0.85 (0.39-1.8)
	High school	61 (4.1)	12 (19.7)	49 (80.3)	0.32 (0.17-.61)
	University	44 (2.9)	13 (29.5)	31 (70.5)	0.55 (0.28-1.07)
occupational status	Unemployed	94 (6.3)	54 (57.4)	40 (42.5)	1
	Housekeeper	591 (39.4)	350 (59.2)	241 (40.8)	1.08 (0.69-1.67)
	Retired	330 (22)	103 (31.2)	227 (68.8)	0.34 (0.21-0.54)
	Free job	476 (31.8)	118 (24.8)	358 (75.2)	0.24 (0.15-0.39)
	undetermined	8 (5)	5 (62.5)	3 (37.5)	1.23 (0.28-5.4)
Diabetes mellitus	Yes	464 (31)	214 (46.1)	250 (53.9)	1.27 (1.02-1.59)
	No	1035 (69)	416 (40.2)	619 (59.8)	1
hypertension	Yes	932 (62.6)	400 (42.9)	539 (57.1)	1.09 (0.88-1.34)
	no	567 (37.8)	230 (40.6)	337 (59.4)	1
Chronic pain	Yes	1258 (83.9)	557 (44.3)	701 (55.7)	1.83 (1.36-2.46)
	No	241 (16.1)	73 (30.3)	168 (69.7)	1
Back pain	Yes	942 (62.8)	479 (50.8)	463 (49.2)	2.72(2.17-3.4)
	No	557 (37.2)	151 (27.1)	406 (72.9)	1
Marital status	Marriage	1281 (85.5)	510 (39.8)	771 (60.2)	1
	Single	218 (14.5)	120 (55)	98 (45)	1.84(1.39-2.47)
Live alone	Yes	102 (6.8)	56 (54.9)	46 (45.1)	1
	no	1397 (93.2)	574 (41.1)	832 (58.9)	1.77(1.18-2.4)
Vitamin D deficiency	yes	76 (5.1)	45 (59.2)	31 (40.8)	1
	no	1423 (94.9)	585 (41.1)	838 (58.9)	2.08(1.3-3.3)
MMSE #	(≤25)	459 (30.6)	243 (52.9)	216 (47.1)	1.9(1.52-2.37)
	>26	1040 (69.4)	387 (37.2)	653 (62.8)	1

@ Chi square test

# MMSE (Mini Mental State Exam)

## Discussion

The findings of this study indicated that 42% of subjects of this cohort had headache and 42.4% had depression. Headache was more prevalent in women than men by odds of 3.83. There was a significant association between headache and depression. The prevalence of headache increased by severity of depression with a dose-response relationship pattern. After adjustment for all clinical and demographic variables headache was independently associated with depression, chronic pain, back pain, number of co morbidities and female gender. Many common demographic and clinical conditions such as hypertension, diabetes, age, had no relation with headache.

Association between headache and psychological factors such as depression, stress as observed in the present study has been reported in other studies as well (6, 8, 9, 12, 13). Uthaikup et al. in a study of 162 individuals aged of 60- 75 years found a direct relationship between depression and headache in the elderly subjects (9). In a study by Rausa et al, 46.8% of patients with daily chronic pain suffered from mental disorders (12). 

In another study by Ruscheweyh et al., the association was more evident in patients with greater than 13 attacks of headache per month (13). In a study of Cohen et al, in 214 white and 859 African American patients with the average age of 68 years, 17.8% suffered from headache and the data analysis revealed a direct connection between levels of stress and depression with headache in older subjects (8). Similarly in a study of 92 patients with chronic headache, 53.3% had psychological disorders (6). A similar association was observed between level of depression and stress with intensity of migraine and tension headache (14, 15).

Nonetheless, the results of one study indicated a relationship between psychological disorders with pain alone rather than headache or migraine (16). This issue was confirmed in another case-control study of patients with chronic recurrent abdominal pain and headache. The authors found an association between headache and abdominal pain but not with psychological problems (17).

In the present study, the prevalence of headaches was significantly higher among patients suffering from cognitive disorders when compared to normal patients. Headache was also more prevalent among subjects with low level of education when compared to those with higher levels of education. Although the association decreased to nonsignificant level after adjustment. Other studies have shown an association between headache and psychological disorders especially among women (18, 19). Information in relation to pain and mental illness may be helpful regarding etiological diagnosis as well as treatment (5). Higher prevalence of headache in depressive subjects may be attributed to greater outward symptoms in depressed patients rather than normal people. It was shown that patients with self-reported depressive symptoms suffer more frequently from chronic pain (5). This might be explainable by considering similar mechanism of pain and mood disorders in central nervous system, possibly involving the limbic system. Lower level of serotonin in depressive state may be associated as well (6).

There has been a significant relationship between self-reported depression and chronic pain such as back pain and headaches. It has been shown that patients aged > 45 years old are more prone to both chronic pain and depressive symptoms (5). 

It should be remembered that many depressed patients do not receive treatment and even a definitive diagnosis of depression may have not been established the association of psychological disorders and chronic headache as observed in our study has been reported in several studies. This issue may be attributed to inadequate ability to combat depressive effects or presence of functional disability as well as inability to take appropriate treatment (6, 7, 8).

The results of this study should be considered with limitations. We did not provide data regarding secondary causes of headache. In the present study chronic pain and low back pain were independently associated with headache. In this geographic region, vitamin D deficiency is highly prevalent (20) and is independently associated with chronic bone pain and back pain as well as skeletal pain (21-23). Restoration of vitamin D deficiency was associated with improvement of skeletal pain (24). 

It was speculated that both musculoskeletal pain and headache may be symptoms of subclinical osteomalacia in vitamin D deficient subjects and in vitamin D deficiency state, headache maybe a symptom of skull bone involvement (25). In any case in the present study vitamin D deficiency was not associated with headache. Overuse of medications for pain relief is a risk factor for chronic pain including headache. 

In one study, 78% of patients presented to a pain clinic were taking analgesic for more than 15 days per month (26). In an earlier study of this cohort, polypharmacy was observed in 34.3% of women with osteoporosis (11, 27) and mean number of medical disease in study patients of the current study was 3.76 (data not shown). Which was significantly associated with headache. In addition several common chronic disease such as diabetes, obesity, metabolic syndrome, hypertension are prevalent in this geographic region and one or more number of these conditions may coexist in study patients (11, 28-30) which require to be managed by taking several drugs. In a study of women with migraine, metabolic syndrome was associated with chronic migraine (31).

Nevertheless, the contribution of these variables in the development of headache can not be ignored because we did not provide data in this context. Regardless, since all study patients were recruited from the same general population with homogeneous ethnic, social, demographic and lifestyle characteristics, thus confounding factors are likely to be distributed similarly across various comparison groups and so, the results expected to confound minimally.

One major limitation of this study is related to study design which is cross sectional, and the association does not indicate causality. Another limitation lies in the fact that data regarding presence or absence of headache is self-reported and its reproducibility may be subjected to bias. Notwithstanding the strength of this study is dependent on patients’ selection which selected all inhabitants of a small town. Therefore the study patients of this study represent general population.

In conclusion the findings of this study indicated high prevalence of headache and depression in elderly subjects aged 60 years and older. There was a significant and independent association between headache and depression, chronic pain, female sex, number of coexisting comorbidities and back pain. Although the nature of this study is case-control and the observed association does not indicate causality, the presence of a significant dose-response relationship between severity of depression with headache indicate in favor of causality. In either way, this issue need a longitudinal study.

## Notes

**Funding:** This research was financially supported by Faculty of Medicine (no: 2139 Babol University of Medical Sciences).
